# Absence of detectable bovine leukemia virus miRNAs in human cancer small RNA-seq datasets

**DOI:** 10.1128/spectrum.03818-25

**Published:** 2026-03-16

**Authors:** Juan Pablo Jaworski

**Affiliations:** 1 Instituto de Virología e Innovaciones Tecnológicas (IVIT), Instituto Nacional de Tecnología Agropecuaria (INTA), Consejo Nacional de Investigaciones Científicas y Tecnológicas (CONICET), Buenos Aires, Argentina; University of Manitoba, Winnipeg, Manitoba, Canada

**Keywords:** micro-RNA, miRNA, cancer, human, bovine leukemia virus, BLV, virus

## Abstract

Bovine leukemia virus (BLV) encodes 10 mature microRNAs (miRNAs) that are highly expressed in infected cattle and have been implicated in oncogenic mechanisms. A potential association between BLV and human cancer remains controversial: while several reports have detected BLV proviral DNA in human breast tissue, high-throughput sequencing analyses have failed to identify BLV nucleic acids in human tumors. Here, we performed a high-sensitivity screen for BLV miRNAs across 335 publicly available human small RNA-seq data sets, including breast cancer (BCA), acute lymphoblastic leukemia, acute myeloid leukemia, chronic lymphocytic leukemia, and healthy controls, totaling approximately 1.98 billion trimmed reads. To validate our analytical workflow, six small RNA data sets from BLV-infected and uninfected cattle were processed in parallel. Across the human data sets, only 60 reads (0.000003%) aligned to BLV miRNA reference sequences, predominantly STAR strands or sequences containing mismatches—patterns consistent with background noise rather than biologically meaningful viral miRNAs. In contrast, BLV miRNAs were robustly detected across BLV-infected cattle samples, confirming pipeline sensitivity for canonical BLV miRNAs. These results demonstrate that BLV miRNAs are absent or below the operational detection threshold in human BCA and human leukemia miRNomes using standard small-RNA sequencing workflows and canonical reference sequences.

**IMPORTANCE:**

Bovine leukemia virus (BLV) causes leukemia in cattle. BLV genome encodes for microRNAs (miRNAs) that can influence how cells grow. Some researchers have suggested that BLV might also play a role in human cancers, especially breast cancer (BCA), but scientific studies have produced conflicting results. In our work, we analyzed nearly two billion genetic sequences from 335 human cancer samples—including BCA and several types of leukemia—to look for BLV miRNAs. We found almost no sequences that resembled BLV miRNAs, and those few likely represented background noise rather than true viral signals. In contrast, BLV miRNAs were easily detected in infected cattle samples. Our results indicate that BLV miRNAs are not present at meaningful levels in particular human cancers (i.e., leukemia and BCA).

## INTRODUCTION

Bovine leukemia virus (BLV) is a deltaretrovirus that causes malignant B-cell lymphoma in up to 10% of infected cattle (1). There are controversies regarding the association between BLV and cancer development in humans (2). In a series of case-control studies, involving samples of breast tissue from the United States (*n* = 224) and Australia (*n* = 96), Buehring et al. reported that the presence of BLV proviral DNA (determined by *in situ* PCR) augmented the risk of developing breast cancer (BCA) in both populations (OR = 3.1 and OR = 4.7, respectively) (3, 4). In addition, several other publications support the association between exposure to BLV and human cancer (5–11). Although BLV-reactive antibodies have been detected in human plasma (12), the potential route of BLV infection in humans is not known. In this regard, the consumption of untreated dairy products from BLV-infected animals has been proposed as a possible one. Another publication by Dr. Buehring reported the presence of BLV in human blood (13). Oppositely, one large study failed to demonstrate any association between BLV and BCA in Chinese patients using different serological and molecular assays (14). Additionally, whole-genome and whole-transcriptome sequencing analysis of BCA samples, obtained from The Cancer Genome Atlas (TCGI, National Center for Biotechnology Information [NCBI]), failed to detect sequencing reads corresponding to proviral DNA or viral transcripts in these samples (15, 16). These conflicting findings prompted interest in exploring alternative viral factors, such as BLV-derived microRNAs (miRNAs), that might contribute to oncogenic processes.

miRNAs are small RNA (sRNA) molecules, typically 19–24 nucleotides long, that bind by complementarity with sequences present in messenger RNAs (mRNA). As a result of this interaction, the target mRNA is degraded, and translation is suppressed (17). It is widely assumed that miRNAs are constitutively dysregulated in different human cancer types, affecting key biological pathways that finally lead to tumor progression (18). Recently, BLV-encoded miRNAs have been associated with the regulation of different genes involved in oncogenic pathways. One of BLV-encoded miRNAs (blv-miR-b4-3p) shares nine nucleotides of its seed region with a miR-29a (19, 20). miR-29a is a well-described oncomiR that targets HMG-box transcription factor 1 (HBP-1) and peroxidasin homolog (PXDN) mRNAs, reducing their expression (19, 20). Both HBP-1 and PXDN gene products have anti-tumoral activity in B cells. Endogenous miR-29a overexpression is linked to B-cell malignancies in humans (21). *In vitro* and *ex vivo* studies showed that blv-miR-b4-3p downregulated the expression of HBP-1 and PXDN (19, 20), and *in vivo* expression of blv-miR-b4-3p was linked to PXDN downregulation in cattle naturally infected with BLV (22). Considering the oncogenic role of miR-29a, it is possible that blv-miR-b4-3p could be involved in BLV-associated tumorigenesis mechanisms.

It is also known that miRNAs can be conveyed through lipid extracellular vesicles (EVs) present in milk and colostrum of humans and cattle (23–25). These miRNAs are refractory to diverse industrial processes (i.e., pasteurization) (26–28). Moreover, the EVs containing miRNAs are stabilized in the gastrointestinal tract, favoring their absorption and subsequent transfer into the bloodstream of the individuals who ingest them (29–32). Furthermore, *in vitro* studies demonstrated (i) the ability of human cells to incorporate exogenous miRNAs from bovine vesicles (30, 33) and (ii) that BLV miRNAs can interact with human genes involved in tumor processes (19, 20). Given these observations, we sought to empirically determine whether BLV miRNAs could be detected in human cancers such as BCA, leukemia, and lymphoma. Rather than testing causation, we aimed to perform a high-sensitivity screen across multiple data sets to assess whether any detectable signal of BLV miRNAs exists in human cancer miRNomes.

## MATERIALS AND METHODS

### Human and animal data sets

We analyzed a total of 335 human miRNomes from nine different small RNAseq open studies from the NCBI (PRJNA758408, PRJNA792999, PRJNA934049, PRJNA1042957, PRJEB52224, PRJNA892942, PRJNA1024943, PRJNA996975, and PRJNA1126751) for the presence of BLV miRNAs. Two hundred twenty-eight samples from cancer patients: BCA, acute lymphoblastic leukemia (ALL), acute myeloid leukemia (AML), chronic lymphocytic leukemia (CLL), and 107 from healthy controls. To validate the detection sensitivity of our workflow, we also analyzed six bovine data sets (three BLV-infected and three uninfected; PRJNA378560) previously characterized for BLV miRNA expression.

### Small RNA sequencing reads processing

All data analyses were performed using the Galaxy platform (version 24.1.4) (https://usegalaxy.org/). Raw fastq files were transferred from NCBI (https://www.ncbi.nlm.nih.gov/sra) to the Galaxy server for downstream analysis. Quality evaluation of the raw sequence data was done with FastQC (Version 0.12.1) (http://www.bioinformatics.babraham.ac.uk/projects/fastqc/). The basic statistics, the sequence quality, quality score, sequence content, and GC content of each raw sequence were evaluated, and raw data that fulfilled basic quality parameters were used for detection analysis. Adapters (i.e., Illumina Universal and Illumina Small Seq) and low-quality ends were trimmed using CutAdapt (version 4.9) (34) and/ or Trim Galore (version 0.6.7) (https://www.bioinformatics.babraham.ac.uk/projects/trim_galore/), and sequences with less than 18 bp or more than 30 bp were removed from downstream analysis. Following this, the fastq files were parsed into fasta format, sequences with non-canonical nucleotides were discarded, and identical reads were collapsed using the miRDeep2 mapper tool (version 2.0.0.8) (35).

### miRNAs identification and quantification

Prior to sequence alignment, all BLV and control miRNAs and hairpin (precursor) reference sequences (Table S1) were downloaded from the miRbase (https://www.mirbase.org/) (36) and uploaded to Galaxy.org in fasta format. We searched for BLV miRNAs in human cancer samples using miRDeep2 quantifier. We deliberately used this tool for the detection and quantification of known BLV miRNAs with maximal sensitivity. We used collapsed sRNA-seq reads and mature and precursor BLV-encoded miRNAs as inputs. miRNA quantification involved first mapping reads to miRNA precursor sequences, followed by mapping to corresponding mature miRNAs. We allowed up to two mismatches to increase sensitivity. Normalization was omitted to avoid losing weak signals, as the study’s aim was not to assess differential expression but rather the presence of BLV miRNAs. Additionally, a set of human miRNAs (hsa-mir-29a-3p, hsa-miR-106b-5p, and hsa-miR-21-5p) served as a positive control for data integrity. CSV files generated by the quantifier module of the miRDeep2 software were downloaded, modified, and shown in Tables 1 to 9.

**TABLE 1:** PRJNA1126751: Small RNA sequencing of exosomes in BCA screening population (human)^*a*^

				Number of reads that align on												
Sample ID	Cancer type	Post trim reads	Total no of reads	blv-miR-B1-3p	blv-miR-B1-5p	blv-miR-B2-3p	blv-miR-B2-5p	blv-miR-B3-3p	blv-miR-B3-5p	blv-miR-B4-3p	blv-miR-B4-5p	blv-miR-B5-3p	blv-miR-B5-5p	hsa-miR-106b-5p	hsa-miR-21-5p	hsa-mir-29a-3p
SRR29496163	NEGATIVE	9.3M	15.0M	0.00	0.00	0.00	0.00	0.00	0.00	0.00	0.00	0.00	0.00	69.00	11,786.00	5331.00
SRR29496164	NEGATIVE	8.4M	13.4M	0.00	0.00	0.00	0.00	0.00	0.00	0.00	0.00	0.00	0.00	29.00	6383.00	2001.00
SRR29496165	NEGATIVE	9.6M	15.1M	0.00	0.00	0.00	0.00	0.00	0.00	0.00	0.00	0.00	0.00	29.00	12,460.00	3044.00
SRR29496166	NEGATIVE	8.3M	13.8M	0.00	1.00	0.00	0.00	0.00	0.00	0.00	0.00	0.00	0.00	9.00	3219.00	859.00
SRR29496167	NEGATIVE	6.7M	10.6M	0.00	0.00	0.00	0.00	0.00	0.00	0.00	0.00	0.00	0.00	19.00	4761.00	2493.00
SRR29496168	NEGATIVE	7.8M	12.5M	0.00	0.00	0.00	0.00	7.00	0.00	0.00	0.00	0.00	0.00	40.00	8136.00	2598.00
SRR29496169	NEGATIVE	7.7M	12.4M	0.00	0.00	0.00	0.00	0.00	0.00	0.00	0.00	0.00	0.00	23.00	8055.00	2020.00
SRR29496170	NEGATIVE	8.3M	13.5M	0.00	0.00	0.00	0.00	0.00	0.00	0.00	0.00	0.00	0.00	22.00	7496.00	3312.00
SRR29496171	NEGATIVE	7.8M	12.7M	0.00	0.00	0.00	0.00	0.00	0.00	0.00	1.00	0.00	0.00	45.00	8004.00	2793.00
SRR29496172	NEGATIVE	10.9M	17.8M	0.00	0.00	0.00	0.00	0.00	0.00	0.00	0.00	0.00	0.00	17.00	4379.00	1665.00
SRR29496173	NEGATIVE	8.4M	13.0M	0.00	0.00	0.00	0.00	0.00	0.00	0.00	0.00	0.00	0.00	77.00	15,023.00	6791.00
SRR29496174	NEGATIVE	8.4M	13.9M	0.00	0.00	0.00	0.00	0.00	0.00	0.00	0.00	0.00	0.00	31.00	8301.00	3669.00
SRR29496175	NEGATIVE	7.5M	12.1M	0.00	0.00	0.00	0.00	0.00	0.00	0.00	0.00	0.00	0.00	53.00	17,808.00	5983.00
SRR29496176	NEGATIVE	7.7M	12.4M	0.00	0.00	0.00	0.00	0.00	0.00	0.00	0.00	0.00	0.00	32.00	12,217.00	2788.00
SRR29496177	NEGATIVE	9.0M	14.3M	0.00	0.00	0.00	0.00	0.00	0.00	0.00	0.00	0.00	0.00	36.00	9733.00	4463.00
SRR29496178	NEGATIVE	13.0M	19.5M	0.00	0.00	0.00	0.00	0.00	0.00	0.00	0.00	0.00	0.00	13.00	10,358.00	1144.00
SRR29496179	NEGATIVE	9.4M	14.7M	0.00	0.00	0.00	0.00	0.00	0.00	0.00	0.00	0.00	0.00	49.00	13,026.00	9079.00
SRR29496180	NEGATIVE	9.2M	14.4M	0.00	0.00	0.00	0.00	0.00	0.00	0.00	0.00	0.00	0.00	61.00	13,765.00	3568.00
SRR29496181	NEGATIVE	8.6M	14.5M	0.00	0.00	0.00	0.00	0.00	0.00	0.00	0.00	0.00	0.00	18.00	2755.00	1067.00
SRR29496182	NEGATIVE	8.9M	14.6M	0.00	0.00	0.00	0.00	0.00	0.00	0.00	0.00	0.00	0.00	23.00	7847.00	1307.00
SRR29496183	NEGATIVE	6.9M	11.2M	0.00	0.00	0.00	0.00	0.00	0.00	0.00	0.00	0.00	2.00	11.00	3005.00	1461.00
SRR29496184	NEGATIVE	10.2M	15.5M	0.00	0.00	0.00	0.00	0.00	0.00	0.00	0.00	0.00	0.00	145.00	21,714.00	5540.00
SRR29496185	NEGATIVE	8.8M	15.1M	0.00	0.00	0.00	0.00	0.00	0.00	0.00	0.00	0.00	0.00	25.00	9108.00	3620.00
SRR29496186	NEGATIVE	6.8M	12.5M	0.00	0.00	0.00	0.00	0.00	0.00	0.00	0.00	0.00	0.00	32.00	11,560.00	3345.00
SRR29496247	NEGATIVE	9.3M	15.9M	0.00	0.00	0.00	0.00	0.00	0.00	0.00	0.00	0.00	0.00	44.00	5744.00	1832.00
SRR29496248	NEGATIVE	7.3M	12.5M	0.00	0.00	0.00	0.00	0.00	0.00	0.00	0.00	0.00	0.00	36.00	6364.00	2662.00
SRR29496249	NEGATIVE	10.8M	16.0M	0.00	0.00	0.00	0.00	0.00	0.00	0.00	0.00	0.00	0.00	88.00	14,772.00	6262.00
SRR29496250	NEGATIVE	9.6M	15.8M	0.00	0.00	0.00	0.00	0.00	0.00	0.00	1.00	0.00	0.00	24.00	4167.00	1985.00
SRR29496251	NEGATIVE	7.6M	12.2M	0.00	0.00	0.00	0.00	0.00	0.00	0.00	0.00	0.00	0.00	22.00	3326.00	1173.00
SRR29496252	NEGATIVE	9.1M	14.1M	0.00	0.00	0.00	0.00	0.00	0.00	0.00	0.00	0.00	0.00	45.00	14,499.00	5077.00
SRR29496253	NEGATIVE	7.6M	12.2M	0.00	0.00	0.00	0.00	0.00	0.00	0.00	0.00	0.00	3.00	33.00	5675.00	2108.00
SRR29496254	NEGATIVE	7.8M	12.2M	0.00	0.00	0.00	0.00	0.00	0.00	0.00	2.00	0.00	0.00	42.00	6571.00	4075.00
SRR29496255	NEGATIVE	11.6M	18.2M	0.00	0.00	0.00	0.00	0.00	0.00	0.00	0.00	0.00	0.00	30.00	9226.00	5788.00
SRR29496256	NEGATIVE	7.6M	12.2M	0.00	0.00	0.00	0.00	0.00	0.00	0.00	0.00	0.00	0.00	53.00	11,636.00	7966.00
SRR29496257	NEGATIVE	9.9M	15.2M	0.00	0.00	0.00	0.00	0.00	0.00	0.00	0.00	0.00	0.00	80.00	17,320.00	6749.00
SRR29496258	NEGATIVE	8.2M	13.4M	0.00	0.00	0.00	0.00	0.00	0.00	0.00	0.00	0.00	0.00	50.00	11,005.00	2757.00
SRR29496259	NEGATIVE	8.6M	15.0M	0.00	0.00	0.00	0.00	0.00	0.00	0.00	0.00	0.00	0.00	20.00	9087.00	2386.00
SRR29496291	NEGATIVE	8.6M	14.0M	0.00	0.00	0.00	0.00	0.00	0.00	0.00	0.00	0.00	0.00	20.00	5939.00	2722.00
SRR29496292	NEGATIVE	7.4M	12.9M	0.00	0.00	0.00	0.00	0.00	0.00	0.00	0.00	0.00	0.00	56.00	6018.00	3140.00
SRR29496293	NEGATIVE	8.5M	14.1M	0.00	0.00	0.00	0.00	0.00	0.00	0.00	0.00	0.00	0.00	58.00	21,033.00	3977.00
SRR29496294	NEGATIVE	8.6M	13.9M	0.00	1.00	0.00	1.00	0.00	0.00	0.00	0.00	0.00	0.00	38.00	6263.00	1745.00
SRR29496295	NEGATIVE	7.8M	12.6M	0.00	0.00	0.00	0.00	0.00	0.00	0.00	0.00	0.00	0.00	39.00	6926.00	1472.00
SRR29496296	NEGATIVE	10.0M	15.7M	0.00	0.00	0.00	0.00	0.00	0.00	0.00	0.00	0.00	0.00	44.00	10,016.00	4194.00
SRR29496297	NEGATIVE	9.5M	14.2M	0.00	0.00	0.00	0.00	0.00	0.00	0.00	0.00	0.00	0.00	69.00	33,096.00	7215.00
SRR29496298	NEGATIVE	7.6M	12.0M	0.00	0.00	0.00	0.00	0.00	0.00	0.00	0.00	0.00	0.00	28.00	6102.00	2521.00
SRR29496299	NEGATIVE	7.7M	13.5M	0.00	0.00	0.00	0.00	0.00	0.00	0.00	0.00	0.00	0.00	39.00	7521.00	2868.00
SRR29496300	NEGATIVE	8.3M	14.6M	0.00	0.00	0.00	0.00	0.00	0.00	0.00	0.00	0.00	0.00	127.00	19,201.00	9511.00
SRR29496301	NEGATIVE	10.7M	19.8M	0.00	1.00	0.00	0.00	0.00	0.00	0.00	0.00	0.00	0.00	47.00	17,131.00	4744.00
SRR29496302	NEGATIVE	6.7M	11.1M	0.00	0.00	0.00	0.00	0.00	0.00	0.00	0.00	0.00	0.00	25.00	3845.00	1369.00
SRR29496303	NEGATIVE	6.9M	13.5M	0.00	0.00	0.00	0.00	0.00	0.00	0.00	0.00	0.00	0.00	26.00	3453.00	3652.00
SRR29496304	NEGATIVE	4.8M	11.9M	0.00	0.00	0.00	0.00	0.00	0.00	0.00	0.00	0.00	0.00	202.00	11,127.00	7007.00
SRR29496305	NEGATIVE	7.7M	14.4M	0.00	0.00	0.00	0.00	0.00	0.00	0.00	0.00	0.00	0.00	20.00	8072.00	4234.00
SRR29496306	NEGATIVE	2.3M	3.9M	0.00	0.00	0.00	0.00	0.00	0.00	0.00	0.00	0.00	0.00	6.00	648.00	384.00
SRR29496307	NEGATIVE	4.6M	7.8M	0.00	0.00	0.00	0.00	0.00	0.00	0.00	0.00	0.00	0.00	3.00	1243.00	688.00
SRR29496308	NEGATIVE	5.7M	9.9M	0.00	0.00	0.00	0.00	0.00	0.00	0.00	3.00	0.00	0.00	14.00	3290.00	1718.00
SRR29496309	NEGATIVE	5.2M	9.9M	0.00	0.00	0.00	0.00	0.00	0.00	0.00	0.00	0.00	0.00	33.00	5334.00	3610.00
SRR29496310	NEGATIVE	5.6M	10.4M	0.00	0.00	0.00	0.00	0.00	0.00	0.00	3.00	0.00	0.00	26.00	3112.00	2289.00
SRR29496311	NEGATIVE	6.1M	10.7M	0.00	0.00	0.00	0.00	0.00	0.00	0.00	0.00	0.00	0.00	23.00	3111.00	2144.00
SRR29496312	NEGATIVE	6.2M	9.3M	0.00	0.00	0.00	0.00	0.00	0.00	0.00	0.00	0.00	0.00	662.00	8506.00	5711.00
SRR29496313	NEGATIVE	4.5M	9.0M	0.00	0.00	0.00	0.00	0.00	0.00	0.00	0.00	0.00	0.00	11.00	5996.00	2144.00
SRR29496314	NEGATIVE	8.7M	14.9M	0.00	0.00	0.00	0.00	0.00	0.00	0.00	0.00	0.00	0.00	18.00	9444.00	1809.00
SRR29496191	BCA	5.2M	9.9M	0.00	0.00	0.00	0.00	0.00	0.00	0.00	2.00	0.00	0.00	13.00	5060.00	3922.00
SRR29496196	BCA	5.3M	9.8M	0.00	0.00	0.00	0.00	0.00	0.00	0.00	0.00	0.00	0.00	56.00	10,323.00	4574.00
SRR29496197	BCA	5.4M	10.6M	0.00	0.00	0.00	0.00	0.00	0.00	0.00	0.00	0.00	0.00	7.00	4455.00	2074.00
SRR29496202	BCA	6.1M	11.8M	0.00	0.00	0.00	0.00	0.00	0.00	0.00	0.00	0.00	0.00	57.00	4946.00	4934.00
SRR29496205	BCA	6.4M	10.5M	0.00	0.00	0.00	0.00	0.00	0.00	0.00	0.00	0.00	0.00	27.00	2975.00	1934.00
SRR29496212	BCA	8.8M	13.7M	0.00	0.00	0.00	0.00	0.00	0.00	0.00	0.00	0.00	0.00	181.00	10,771.00	7760.00
SRR29496215	BCA	7.1M	11.3M	0.00	0.00	0.00	0.00	0.00	0.00	0.00	0.00	0.00	0.00	56.00	14,010.00	6758.00

^*a*^ BCA: breast cancer.

**TABLE 2:** PRJNA996975: Profiling of miRNA in pediatric AML in the Egyptian population^*a*^

				Number of reads that align on												
Sample ID	Cancer type	Post trim reads	Total no of reads	blv-miR-B1-3p	blv-miR-B1-5p	blv-miR-B2-3p	blv-miR-B2-5p	blv-miR-B3-3p	blv-miR-B3-5p	blv-miR-B4-3p	blv-miR-B4-5p	blv-miR-B5-3p	blv-miR-B5-5p	hsa-miR-106b-5p	hsa-miR-21-5p	hsa-mir-29a-3p
SRR25374631	NEGATIVE	1.2M	1.2M	0.00	0.00	0.00	0.00	0.00	0.00	0.00	0.00	0.00	0.00	0.00	1.00	0.00
SRR25374632	NEGATIVE	0.3M	0.3M	0.00	0.00	0.00	0.00	0.00	0.00	0.00	0.00	0.00	0.00	1.00	0.00	0.00
SRR25374633	NEGATIVE	1.5M	1.8M	0.00	0.00	0.00	0.00	0.00	0.00	0.00	0.00	0.00	0.00	0.00	3.00	0.00
SRR25374634	AML	1.3M	1.3M	0.00	0.00	0.00	0.00	0.00	0.00	0.00	0.00	0.00	0.00	4.00	63.00	1.00
SRR25374635	AML	1.2M	1.2M	0.00	0.00	0.00	0.00	0.00	0.00	0.00	0.00	0.00	0.00	3.00	70.00	0.00
SRR25374636	AML	1.0M	1.0M	0.00	0.00	0.00	0.00	0.00	0.00	0.00	0.00	0.00	0.00	3.00	44.00	0.00
SRR25374637	AML	0.7M	0.8M	0.00	0.00	0.00	0.00	0.00	0.00	0.00	0.00	0.00	0.00	3.00	7.00	0.00
SRR25374638	AML	0.8M	0.9M	0.00	0.00	0.00	0.00	0.00	0.00	0.00	0.00	0.00	0.00	3.00	22.00	0.00
SRR25374639	AML	1.0M	1.1M	0.00	0.00	0.00	0.00	0.00	0.00	0.00	0.00	0.00	0.00	0.00	95.00	0.00
SRR25374640	AML	0.8M	0.9M	0.00	0.00	0.00	0.00	0.00	0.00	0.00	0.00	0.00	0.00	0.00	11.00	0.00
SRR25374641	AML	0.6M	0.6M	0.00	0.00	0.00	0.00	0.00	0.00	0.00	0.00	0.00	0.00	0.00	19.00	0.00
SRR25374642	AML	0.8M	0.9M	0.00	0.00	0.00	0.00	0.00	0.00	0.00	0.00	0.00	0.00	1.00	14.00	0.00
SRR25374643	AML	1.0M	1.0M	0.00	0.00	0.00	0.00	0.00	0.00	0.00	0.00	0.00	0.00	1.00	28.00	0.00
SRR25374644	AML	1.0M	1.1M	0.00	0.00	0.00	0.00	0.00	0.00	0.00	0.00	0.00	0.00	6.00	148.00	1.00
SRR25374645	AML	1.1M	1.2M	0.00	0.00	0.00	0.00	0.00	0.00	0.00	0.00	0.00	0.00	1.00	108.00	0.00
SRR25374646	AML	0.8M	0.8M	0.00	0.00	0.00	0.00	0.00	0.00	0.00	0.00	0.00	0.00	5.00	9.00	0.00
SRR25374647	AML	0.8M	0.9M	0.00	0.00	0.00	0.00	0.00	0.00	0.00	0.00	0.00	0.00	0.00	35.00	0.00
SRR25374648	AML	0.9M	1.0M	0.00	0.00	0.00	0.00	0.00	0.00	0.00	0.00	0.00	0.00	0.00	29.00	0.00
SRR25374649	AML	1.2M	1.2M	0.00	0.00	0.00	0.00	0.00	0.00	0.00	0.00	0.00	0.00	1.00	77.00	0.00
SRR25374650	AML	0.9M	0.9M	0.00	0.00	0.00	0.00	0.00	0.00	0.00	0.00	0.00	0.00	6.00	44.00	0.00
SRR25374651	AML	0.3M	0.3M	0.00	0.00	0.00	0.00	0.00	0.00	0.00	0.00	0.00	0.00	0.00	1.00	0.00
SRR25374652	AML	0.3M	0.3M	0.00	0.00	0.00	0.00	0.00	0.00	0.00	0.00	0.00	0.00	1.00	17.00	0.00
SRR25374653	AML	0.8M	0.8M	0.00	0.00	0.00	0.00	0.00	0.00	0.00	0.00	0.00	0.00	1.00	27.00	0.00
SRR25374654	AML	0.9M	0.9M	0.00	0.00	0.00	0.00	0.00	0.00	0.00	0.00	0.00	0.00	0.00	11.00	0.00
SRR25374655	AML	1.0M	1.0M	0.00	0.00	0.00	0.00	0.00	0.00	0.00	0.00	0.00	0.00	1.00	10.00	0.00
SRR25374656	AML	0.9M	0.9M	0.00	0.00	0.00	0.00	0.00	0.00	0.00	0.00	0.00	0.00	2.00	65.00	0.00
SRR25374657	AML	0.6M	0.7M	0.00	0.00	0.00	0.00	0.00	0.00	0.00	0.00	0.00	0.00	1.00	61.00	0.00
SRR25374658	AML	0.7M	0.7M	0.00	0.00	0.00	0.00	0.00	0.00	0.00	0.00	0.00	0.00	1.00	4.00	0.00
SRR25374659	AML	0.9M	0.9M	0.00	0.00	0.00	0.00	0.00	0.00	0.00	0.00	0.00	0.00	0.00	68.00	0.00
SRR25374660	AML	0.7M	0.7M	0.00	0.00	0.00	0.00	0.00	0.00	0.00	0.00	0.00	0.00	6.00	175.00	0.00
SRR25374661	AML	0.9M	0.9M	0.00	0.00	0.00	0.00	0.00	0.00	0.00	0.00	0.00	0.00	2.00	28.00	0.00
SRR25374662	AML	0.8M	0.9M	0.00	0.00	0.00	0.00	0.00	0.00	0.00	1.00	0.00	0.00	4.00	75.00	0.00
SRR25374663	AML	1.4M	1.6M	0.00	0.00	0.00	0.00	0.00	0.00	0.00	0.00	0.00	0.00	7.00	261.00	0.00
SRR25374664	AML	0.9M	1.0M	0.00	0.00	0.00	0.00	0.00	0.00	0.00	0.00	0.00	0.00	2.00	14.00	0.00
SRR25374665	AML	1.3M	1.4M	0.00	0.00	0.00	0.00	0.00	0.00	0.00	0.00	0.00	0.00	7.00	62.00	0.00
SRR25374666	AML	0.8M	0.8M	0.00	0.00	0.00	0.00	0.00	0.00	0.00	0.00	0.00	0.00	3.00	31.00	0.00
SRR25374667	AML	1.6M	1.7M	0.00	0.00	0.00	0.00	0.00	0.00	0.00	0.00	0.00	0.00	3.00	84.00	0.00
SRR25374668	AML	0.5M	0.6M	0.00	0.00	0.00	0.00	0.00	0.00	0.00	0.00	0.00	0.00	0.00	4.00	0.00
SRR25374669	AML	1.5M	1.6M	0.00	0.00	0.00	0.00	0.00	0.00	0.00	0.00	0.00	0.00	8.00	163.00	0.00
SRR25374670	AML	1.3M	1.4M	0.00	0.00	0.00	0.00	0.00	0.00	0.00	1.00	0.00	0.00	0.00	16.00	0.00
SRR25374671	AML	1.5M	1.5M	0.00	0.00	0.00	0.00	0.00	0.00	0.00	0.00	0.00	0.00	4.00	23.00	0.00
SRR25374672	AML	1.5M	1.6M	0.00	0.00	0.00	0.00	0.00	0.00	0.00	0.00	0.00	0.00	5.00	267.00	0.00
SRR25374673	AML	0.9M	1.0M	0.00	0.00	0.00	0.00	0.00	0.00	0.00	0.00	0.00	0.00	2.00	30.00	0.00
SRR25374674	AML	0.6M	0.6M	0.00	0.00	0.00	0.00	0.00	0.00	0.00	0.00	0.00	0.00	0.00	9.00	0.00
SRR25374675	AML	0.8M	0.8M	0.00	0.00	0.00	0.00	0.00	0.00	0.00	0.00	0.00	0.00	6.00	17.00	0.00
SRR25374676	AML	0.6M	0.8M	0.00	0.00	0.00	0.00	0.00	0.00	0.00	0.00	0.00	0.00	3.00	67.00	0.00
SRR25374677	AML	0.7M	0.8M	0.00	0.00	0.00	0.00	0.00	0.00	0.00	0.00	0.00	0.00	0.00	116.00	0.00
SRR25374678	AML	1.1M	1.1M	0.00	0.00	0.00	0.00	0.00	0.00	0.00	0.00	0.00	0.00	5.00	50.00	0.00
SRR25374679	AML	1.2M	1.3M	0.00	0.00	0.00	0.00	0.00	0.00	0.00	0.00	0.00	0.00	10.00	130.00	0.00
SRR25374680	AML	0.8M	0.8M	0.00	0.00	0.00	0.00	0.00	0.00	0.00	1.00	0.00	0.00	0.00	50.00	0.00
SRR25374681	AML	0.9M	0.9M	0.00	0.00	0.00	0.00	0.00	0.00	0.00	0.00	0.00	0.00	6.00	169.00	0.00
SRR25374682	AML	1.1M	1.1M	0.00	0.00	0.00	0.00	0.00	0.00	0.00	0.00	0.00	0.00	5.00	342.00	0.00
SRR25374683	AML	1.0M	1.1M	0.00	0.00	0.00	0.00	0.00	0.00	0.00	0.00	0.00	0.00	2.00	24.00	0.00
SRR25374684	AML	0.7M	0.7M	0.00	0.00	0.00	0.00	0.00	0.00	0.00	0.00	0.00	0.00	5.00	32.00	0.00
SRR25374685	AML	0.7M	0.7M	0.00	0.00	0.00	0.00	0.00	0.00	0.00	0.00	0.00	0.00	4.00	40.00	0.00
SRR25374686	AML	0.9M	0.9M	0.00	0.00	0.00	0.00	0.00	0.00	0.00	0.00	0.00	0.00	7.00	106.00	2.00
SRR25374687	AML	0.8M	0.9M	0.00	0.00	0.00	0.00	0.00	0.00	0.00	0.00	0.00	0.00	1.00	37.00	0.00
SRR25374688	AML	0.8M	0.9M	0.00	0.00	0.00	0.00	0.00	0.00	0.00	0.00	0.00	0.00	4.00	27.00	0.00
SRR25374689	AML	0.9M	0.9M	0.00	0.00	0.00	0.00	0.00	0.00	0.00	0.00	0.00	0.00	3.00	279.00	0.00
SRR25374690	AML	1.1M	1.2M	0.00	1.00	0.00	0.00	0.00	0.00	0.00	0.00	0.00	0.00	9.00	108.00	0.00
SRR25374691	AML	0.5M	0.6M	0.00	0.00	0.00	0.00	0.00	0.00	0.00	0.00	0.00	0.00	0.00	28.00	0.00
SRR25374692	AML	1.3M	1.3M	0.00	0.00	0.00	0.00	0.00	0.00	0.00	0.00	0.00	0.00	11.00	141.00	0.00
SRR25374693	AML	1.1M	1.1M	0.00	0.00	0.00	0.00	0.00	0.00	0.00	0.00	0.00	0.00	4.00	29.00	0.00
SRR25374694	AML	1.6M	1.6M	0.00	0.00	0.00	0.00	0.00	0.00	0.00	0.00	0.00	0.00	26.00	117.00	0.00
SRR25374695	AML	0.9M	0.9M	0.00	0.00	0.00	0.00	0.00	0.00	0.00	0.00	0.00	0.00	1.00	23.00	0.00
SRR25374696	AML	1.4M	1.4M	0.00	0.00	0.00	0.00	0.00	0.00	0.00	0.00	0.00	0.00	5.00	31.00	0.00
SRR25374697	AML	1.4M	1.4M	0.00	0.00	0.00	0.00	0.00	0.00	0.00	0.00	0.00	0.00	1.00	13.00	0.00
SRR25374698	AML	1.0M	1.0M	0.00	0.00	0.00	0.00	0.00	0.00	0.00	0.00	0.00	0.00	2.00	117.00	0.00
SRR25374699	AML	0.7M	1.3M	0.00	0.00	0.00	0.00	0.00	0.00	0.00	0.00	0.00	0.00	0.00	67.00	0.00
SRR25374700	AML	1.0M	1.2M	0.00	0.00	0.00	0.00	0.00	0.00	0.00	0.00	0.00	0.00	1.00	17.00	0.00
SRR25374701	AML	1.4M	1.5M	0.00	0.00	0.00	0.00	0.00	0.00	0.00	0.00	0.00	0.00	1.00	72.00	1.00
SRR25374702	AML	1.4M	1.4M	0.00	0.00	0.00	0.00	0.00	0.00	0.00	0.00	0.00	0.00	5.00	223.00	1.00
SRR25374703	AML	1.3M	1.3M	0.00	0.00	0.00	0.00	0.00	0.00	0.00	0.00	0.00	0.00	8.00	80.00	0.00
SRR25374704	AML	1.4M	1.5M	0.00	0.00	0.00	0.00	0.00	0.00	0.00	0.00	0.00	0.00	0.00	493.00	0.00
SRR25374705	AML	1.5M	1.6M	0.00	0.00	0.00	0.00	0.00	0.00	0.00	0.00	0.00	0.00	1.00	55.00	0.00
SRR25374706	AML	1.7M	1.7M	0.00	0.00	0.00	0.00	0.00	0.00	0.00	0.00	0.00	0.00	0.00	50.00	0.00
SRR25374707	AML	0.6M	0.6M	0.00	0.00	0.00	0.00	0.00	0.00	0.00	0.00	0.00	0.00	0.00	6.00	0.00
SRR25374708	AML	1.2M	1.2M	0.00	0.00	0.00	0.00	0.00	0.00	0.00	0.00	0.00	0.00	2.00	241.00	0.00
SRR25374709	AML	1.3M	1.4M	0.00	0.00	0.00	0.00	0.00	0.00	0.00	0.00	0.00	0.00	11.00	313.00	0.00
SRR25374710	AML	1.5M	1.6M	0.00	0.00	0.00	0.00	0.00	0.00	0.00	0.00	0.00	0.00	6.00	285.00	0.00
SRR25374711	AML	1.4M	1.4M	0.00	0.00	0.00	0.00	0.00	0.00	0.00	0.00	0.00	0.00	1.00	2.00	0.00
SRR25374712	AML	1.2M	1.3M	0.00	0.00	0.00	0.00	0.00	0.00	0.00	0.00	0.00	0.00	2.00	17.00	0.00
SRR25374713	AML	1.2M	1.2M	0.00	0.00	0.00	0.00	0.00	0.00	0.00	0.00	0.00	0.00	0.00	42.00	0.00
SRR25374714	AML	1.4M	1.6M	0.00	0.00	0.00	0.00	0.00	0.00	0.00	0.00	0.00	0.00	0.00	131.00	0.00
SRR25374715	AML	1.5M	1.6M	0.00	0.00	0.00	0.00	0.00	0.00	0.00	0.00	0.00	0.00	1.00	139.00	0.00
SRR25374716	AML	0.9M	1.0M	0.00	0.00	0.00	0.00	0.00	0.00	0.00	0.00	0.00	0.00	0.00	26.00	0.00
SRR25374717	AML	0.7M	0.7M	0.00	0.00	0.00	0.00	0.00	0.00	0.00	0.00	0.00	0.00	0.00	37.00	0.00
SRR25374718	AML	0.4M	0.4M	0.00	0.00	0.00	0.00	0.00	0.00	0.00	0.00	0.00	0.00	0.00	15.00	0.00
SRR25374719	AML	0.8M	0.9M	0.00	0.00	0.00	0.00	0.00	0.00	0.00	1.00	0.00	0.00	1.00	9.00	0.00
SRR25374720	AML	0.7M	0.7M	0.00	0.00	0.00	0.00	0.00	0.00	0.00	0.00	0.00	0.00	1.00	24.00	1.00
SRR25374721	AML	0.6M	0.7M	0.00	0.00	0.00	0.00	0.00	0.00	0.00	0.00	0.00	0.00	3.00	14.00	0.00
SRR25374722	AML	0.9M	0.9M	0.00	0.00	0.00	0.00	0.00	0.00	0.00	0.00	0.00	0.00	0.00	68.00	0.00
SRR25374723	AML	0.9M	0.9M	0.00	0.00	0.00	0.00	0.00	0.00	0.00	0.00	0.00	0.00	1.00	13.00	0.00
SRR25374724	AML	0.9M	0.9M	0.00	0.00	0.00	0.00	0.00	0.00	0.00	0.00	0.00	0.00	0.00	11.00	0.00
SRR25374725	AML	1.0M	1.1M	0.00	0.00	0.00	0.00	0.00	0.00	0.00	0.00	0.00	0.00	0.00	7.00	0.00
SRR25374726	AML	0.4M	0.4M	0.00	0.00	0.00	0.00	0.00	0.00	0.00	0.00	0.00	0.00	3.00	27.00	0.00
SRR25374727	AML	0.7M	0.7M	0.00	0.00	0.00	0.00	0.00	0.00	0.00	0.00	0.00	0.00	0.00	52.00	0.00
SRR25374728	NEGATIVE	1.1M	1.2M	0.00	0.00	0.00	0.00	0.00	0.00	0.00	0.00	0.00	0.00	0.00	8.00	0.00
SRR25374729	AML	0.9M	0.9M	0.00	0.00	0.00	0.00	0.00	0.00	0.00	0.00	0.00	0.00	1.00	413.00	0.00
SRR25374730	AML	0.5M	0.5M	0.00	0.00	0.00	0.00	0.00	0.00	0.00	0.00	0.00	0.00	1.00	12.00	0.00
SRR25374731	AML	0.7M	0.7M	0.00	0.00	0.00	0.00	0.00	0.00	0.00	0.00	0.00	0.00	1.00	228.00	0.00

^*a*^ AML: acute myeloid leukemia.

**TABLE 3:** PRJNA1024943: Small RNA-seq reveals key miRNAs involved in cell differentiation arrest in AML^*a*^^,^^*b*^

				Number of reads that align on												
Sample ID	Cancer type	Post trim reads	Total no of reads	blv-miR-B1-3p	blv-miR-B1-5p	blv-miR-B2-3p	blv-miR-B2-5p	blv-miR-B3-3p	blv-miR-B3-5p	blv-miR-B4-3p	blv-miR-B4-5p	blv-miR-B5-3p	blv-miR-B5-5p	hsa-miR-106b-5p	hsa-miR-21-5p	hsa-mir-29a-3p
SRR26304152	AML CR	9.1M	9.9M	0.00	0.00	0.00	0.00	0.00	0.00	0.00	1.00	0.00	0.00	3922.00	92,147.00	5473.00
SRR26304153	AML CR	7.0M	10.0M	0.00	0.00	0.00	0.00	0.00	0.00	0.00	0.00	0.00	0.00	4064.00	171,965.00	41,921.00
SRR26304154	AML CR	8.3M	12.9M	0.00	1.00	0.00	0.00	0.00	0.00	0.00	0.00	0.00	0.00	4769.00	302,613.00	40,566.00
SRR26304155	AML CR	6.6M	8.9M	0.00	0.00	0.00	0.00	0.00	0.00	0.00	0.00	0.00	0.00	4930.00	226,499.00	45,406.00
SRR26304156	AML CR	5.4M	9.2M	0.00	0.00	0.00	0.00	0.00	0.00	0.00	0.00	0.00	0.00	2683.00	165,299.00	22,155.00
SRR26304157	AML ID	6.9M	8.4M	0.00	0.00	0.00	0.00	0.00	0.00	0.00	0.00	0.00	0.00	3986.00	124,081.00	15,695.00
SRR26304158	AML ID	10.4M	12.3M	0.00	0.00	0.00	0.00	0.00	0.00	0.00	0.00	0.00	0.00	9171.00	144,842.00	30,344.00
SRR26304159	AML ID	7.1M	9.2M	0.00	0.00	0.00	0.00	0.00	0.00	0.00	0.00	0.00	0.00	6178.00	69,138.00	13,496.00
SRR26304160	AML ID	7.3M	8.2M	0.00	0.00	0.00	0.00	0.00	0.00	0.00	0.00	0.00	0.00	4175.00	271,174.00	37,096.00
SRR26304161	AML ID	10.6M	15.1M	0.00	0.00	0.00	0.00	0.00	0.00	0.00	0.00	0.00	0.00	8840.00	201,785.00	42,485.00

^*a*^ AML CR: acute myeloid leukemia—complete remission.

^*b*^ AML ID: acute myeloid leukemia—initial diagnosis.

**TABLE 4:** PRJNA892942: HDAC1 regulates the chromatin landscape to control transcriptional dependencies in CLL^*a*^

				Number of reads that align on												
Sample ID	Cancer type	Post trim reads	Total no of reads	blv-miR-B1-3p	blv-miR-B1-5p	blv-miR-B2-3p	blv-miR-B2-5p	blv-miR-B3-3p	blv-miR-B3-5p	blv-miR-B4-3p	blv-miR-B4-5p	blv-miR-B5-3p	blv-miR-B5-5p	hsa-miR-106b-5p	hsa-miR-21-5p	hsa-mir-29a-3p
SRR22000574	CLL	11.4M	11.4M	0.00	0.00	0.00	0.00	0.00	0.00	0.00	0.00	0.00	0.00	2128.00	1,223,652.00	206,950.00
SRR22000575	CLL	11.0M	11.0M	0.00	0.00	0.00	0.00	0.00	0.00	0.00	0.00	0.00	0.00	1666.00	451,575.00	129,685.00
SRR22000576	CLL	10.8M	10.8M	0.00	0.00	0.00	0.00	0.00	0.00	0.00	0.00	0.00	0.00	1418.00	1,080,412.00	148,536.00
SRR22000577	CLL	14.5M	14.5M	0.00	0.00	0.00	0.00	0.00	0.00	0.00	0.00	0.00	0.00	2676.00	616,703.00	211,004.00
SRR22000578	CLL	17.7M	17.7M	0.00	0.00	0.00	0.00	0.00	0.00	0.00	0.00	0.00	0.00	2685.00	1,450,078.00	139,692.00
SRR22000579	CLL	20.5M	20.5M	0.00	0.00	0.00	0.00	0.00	0.00	0.00	0.00	0.00	0.00	3777.00	711,561.00	161,183.00
SRR22000580	CLL	8.5M	8.5M	0.00	0.00	0.00	0.00	0.00	0.00	0.00	0.00	0.00	0.00	1608.00	346,386.00	65,391.00
SRR22000581	CLL	9.5M	9.5M	0.00	0.00	0.00	0.00	0.00	0.00	0.00	0.00	0.00	0.00	1182.00	242,849.00	40,059.00
SRR22000582	CLL	7.6M	7.6M	0.00	0.00	0.00	0.00	0.00	0.00	0.00	0.00	0.00	0.00	935.00	314,786.00	58,362.00
SRR22000583	CLL	4.2M	4.2M	0.00	0.00	0.00	0.00	0.00	0.00	0.00	0.00	0.00	0.00	839.00	128,651.00	59,216.00
SRR22000584	CLL	12.1M	12.1M	0.00	0.00	0.00	0.00	0.00	0.00	0.00	0.00	0.00	0.00	1133.00	487,541.00	62,189.00
SRR22000585	CLL	12.4M	12.4M	0.00	0.00	0.00	0.00	0.00	0.00	0.00	0.00	0.00	0.00	2011.00	403,692.00	81,572.00
SRR22000586	CLL	14.5M	14.5M	0.00	0.00	0.00	0.00	0.00	0.00	0.00	0.00	0.00	0.00	2279.00	471,368.00	206,998.00
SRR22000587	CLL	18.7M	18.7M	0.00	0.00	0.00	0.00	0.00	0.00	0.00	0.00	0.00	0.00	2199.00	362,456.00	178,743.00
SRR22000588	CLL	6.7M	6.7M	0.00	0.00	0.00	0.00	0.00	0.00	0.00	0.00	0.00	0.00	1302.00	513,670.00	81,056.00
SRR22000589	CLL	8.5M	8.5M	0.00	0.00	0.00	0.00	0.00	0.00	0.00	0.00	0.00	0.00	777.00	225,901.00	54,010.00
SRR22000590	CLL	11.4M	11.4M	0.00	0.00	0.00	0.00	0.00	0.00	0.00	0.00	0.00	0.00	2136.00	520,223.00	210,896.00
SRR22000591	CLL	14.2M	14.2M	0.00	2.00	0.00	0.00	0.00	0.00	0.00	0.00	0.00	0.00	1748.00	414,372.00	155,670.00
SRR22000592	CLL	9.1M	9.1M	0.00	0.00	0.00	0.00	0.00	0.00	0.00	0.00	0.00	0.00	1978.00	428,631.00	99,983.00
SRR22000593	CLL	13.4M	13.4M	0.00	0.00	0.00	0.00	0.00	0.00	0.00	0.00	0.00	0.00	2986.00	240,818.00	145,067.00

^*a*^ CLL: chronic lymphocytic leukemia.

**TABLE 5:** PRJEB52224: miRNA-sequencing of Brazilian pediatric ALL^*a*^

				Number of reads that align on												
Sample ID	Cancer type	Post trim reads	Total no of reads	blv-miR-B1-3p	blv-miR-B1-5p	blv-miR-B2-3p	blv-miR-B2-5p	blv-miR-B3-3p	blv-miR-B3-5p	blv-miR-B4-3p	blv-miR-B4-5p	blv-miR-B5-3p	blv-miR-B5-5p	hsa-miR-106b-5p	hsa-miR-21-5p	hsa-mir-29a-3p
ERR9566615	B-cell ALL	7.4M	8.7M	0.00	0.00	0.00	0.00	0.00	0.00	0.00	0.00	0.00	1.00	4249.00	29,214.00	3955.00
ERR9566616	B-cell ALL	7.4M	8.8M	0.00	0.00	0.00	0.00	0.00	0.00	0.00	0.00	0.00	0.00	4305.00	29,472.00	4078.00
ERR9566617	B-cell ALL	6.8M	8.2M	0.00	0.00	0.00	0.00	0.00	0.00	0.00	0.00	0.00	0.00	2752.00	31,394.00	4517.00
ERR9566618	B-cell ALL	6.8M	8.2M	0.00	0.00	0.00	0.00	0.00	0.00	0.00	1.00	0.00	0.00	2783.00	31,711.00	4575.00
ERR9566619	T-cell ALL	0.7M	0.8M	0.00	0.00	0.00	0.00	0.00	0.00	0.00	0.00	0.00	0.00	35.00	1288.00	118.00
ERR9566620	T-cell ALL	0.7M	0.8M	0.00	0.00	0.00	0.00	0.00	0.00	0.00	0.00	0.00	0.00	35.00	1295.00	94.00
ERR9566621	B-cell ALL	8.6M	9.2M	0.00	0.00	0.00	0.00	0.00	0.00	0.00	0.00	0.00	0.00	8594.00	29,879.00	4650.00
ERR9566622	B-cell ALL	8.6M	9.2M	0.00	0.00	0.00	0.00	0.00	0.00	0.00	0.00	0.00	0.00	8262.00	30,113.00	4712.00
ERR9566623	B-cell ALL	5.8M	6.4M	0.00	0.00	0.00	0.00	0.00	0.00	0.00	0.00	0.00	0.00	2529.00	40,542.00	2914.00
ERR9566624	B-cell ALL	5.8M	6.3M	0.00	0.00	0.00	0.00	0.00	0.00	0.00	0.00	0.00	0.00	2578.00	39,850.00	2891.00
ERR9566625	B-cell ALL	6.8M	7.4M	0.00	0.00	0.00	0.00	0.00	0.00	0.00	0.00	0.00	0.00	1294.00	27,291.00	5522.00
ERR9566626	B-cell ALL	6.9M	7.5M	0.00	0.00	0.00	0.00	0.00	0.00	0.00	0.00	0.00	0.00	1342.00	27,457.00	5701.00
ERR9566627	B-cell ALL	9.5M	10.3M	0.00	0.00	0.00	0.00	0.00	0.00	0.00	0.00	0.00	0.00	7039.00	31,058.00	5159.00
ERR9566628	B-cell ALL	9.5M	10.4M	0.00	0.00	0.00	0.00	0.00	0.00	0.00	0.00	0.00	0.00	6850.00	31,330.00	5247.00
ERR9566629	T-cell ALL	4.7M	5.8M	0.00	0.00	0.00	0.00	0.00	0.00	0.00	0.00	0.00	0.00	2487.00	23,154.00	2433.00
ERR9566630	T-cell ALL	4.7M	5.8M	0.00	0.00	0.00	0.00	0.00	0.00	0.00	0.00	0.00	0.00	2507.00	22,762.00	2318.00
ERR9566631	T-cell ALL	4.8M	5.2M	0.00	0.00	0.00	0.00	0.00	0.00	0.00	0.00	0.00	0.00	2837.00	17,828.00	2853.00
ERR9566632	T-cell ALL	4.8M	5.2M	0.00	0.00	0.00	0.00	0.00	0.00	0.00	0.00	0.00	0.00	2669.00	17,277.00	2847.00
ERR9566633	T-cell ALL	3.1M	3.4M	0.00	0.00	0.00	0.00	0.00	0.00	0.00	0.00	0.00	0.00	1486.00	10,149.00	2155.00
ERR9566634	T-cell ALL	3.1M	3.5M	0.00	0.00	0.00	0.00	0.00	0.00	0.00	0.00	0.00	0.00	1416.00	10,213.00	2275.00
ERR9566635	T-cell ALL	7.3M	9.9M	0.00	0.00	0.00	0.00	0.00	0.00	0.00	0.00	0.00	0.00	3681.00	56,502.00	7791.00
ERR9566636	T-cell ALL	7.3M	9.8M	0.00	0.00	0.00	0.00	0.00	0.00	0.00	0.00	0.00	0.00	3709.00	55,543.00	7730.00
ERR9566637	B-cell ALL	5.3M	5.7M	0.00	0.00	0.00	0.00	0.00	0.00	0.00	0.00	0.00	0.00	1594.00	7260.00	1853.00
ERR9566638	B-cell ALL	5.4M	5.7M	0.00	0.00	0.00	0.00	0.00	0.00	0.00	0.00	0.00	0.00	1520.00	7340.00	1890.00
ERR9566639	T-cell ALL	4.8M	7.2M	0.00	0.00	0.00	0.00	0.00	0.00	0.00	0.00	0.00	0.00	1209.00	15,926.00	1057.00
ERR9566640	T-cell ALL	4.8M	7.3M	0.00	0.00	0.00	0.00	0.00	0.00	1.00	0.00	0.00	0.00	1222.00	16,226.00	1117.00
ERR9566641	T-cell ALL	5.9M	6.7M	0.00	0.00	0.00	0.00	0.00	0.00	0.00	0.00	0.00	0.00	1807.00	79,719.00	2335.00
ERR9566642	T-cell ALL	6.0M	6.7M	0.00	0.00	0.00	0.00	0.00	0.00	0.00	0.00	0.00	0.00	1738.00	81,115.00	2366.00
ERR9566643	B-cell ALL	4.1M	4.5M	0.00	0.00	0.00	0.00	0.00	0.00	0.00	0.00	0.00	0.00	586.00	8484.00	1277.00
ERR9566644	B-cell ALL	4.1M	4.5M	0.00	0.00	0.00	0.00	0.00	0.00	0.00	0.00	0.00	0.00	606.00	8367.00	1297.00

^*a*^ ALL: acute lymphoblastic leukemia.

**TABLE 6:** PRJNA1042957: The miRNA expression of 5 normal breast tissues and 15 BCA tissues^*a*^

				Number of reads that align on												
Sample ID	Cancer type	Post trim reads	Total no of reads	blv-miR-B1-3p	blv-miR-B1-5p	blv-miR-B2-3p	blv-miR-B2-5p	blv-miR-B3-3p	blv-miR-B3-5p	blv-miR-B4-3p	blv-miR-B4-5p	blv-miR-B5-3p	blv-miR-B5-5p	hsa-miR-106b-5p	hsa-miR-21-5p	hsa-mir-29a-3p
SRR26902945	BCA	9.3M	11.9M	0.00	0.00	0.00	0.00	0.00	0.00	1.00	0.00	0.00	0.00	1865.00	412,296.00	17,420.00
SRR26902946	BCA	9.1M	11.6M	0.00	0.00	0.00	0.00	0.00	0.00	0.00	0.00	0.00	0.00	2582.00	545,772.00	20,804.00
SRR26902947	BCA	10.9M	12.7M	0.00	0.00	0.00	0.00	0.00	0.00	0.00	0.00	0.00	0.00	1972.00	1,199,853.00	16,764.00
SRR26902948	BCA	10.3M	12.0M	0.00	0.00	0.00	0.00	0.00	0.00	0.00	0.00	0.00	0.00	1372.00	729,057.00	10,757.00
SRR26902949	BCA	9.1M	12.6M	0.00	0.00	0.00	0.00	0.00	0.00	0.00	0.00	0.00	1.00	1040.00	692,073.00	17,156.00
SRR26902950	NEGATIVE	11.3M	13.0M	0.00	0.00	0.00	0.00	0.00	0.00	0.00	0.00	0.00	0.00	1545.00	128,465.00	29,956.00
SRR26902951	NEGATIVE	12.4M	13.8M	0.00	0.00	0.00	0.00	0.00	0.00	0.00	0.00	0.00	0.00	2254.00	111,565.00	16,544.00
SRR26902952	NEGATIVE	11.7M	12.9M	0.00	0.00	0.00	0.00	0.00	0.00	0.00	0.00	0.00	0.00	1072.00	84,363.00	29,109.00
SRR26902953	BCA	9.8M	14.0M	0.00	0.00	0.00	0.00	0.00	0.00	0.00	0.00	0.00	1.00	3683.00	584,070.00	6014.00
SRR26902954	BCA	10.4M	14.1M	0.00	0.00	0.00	0.00	0.00	0.00	0.00	0.00	0.00	0.00	7518.00	877,847.00	48,694.00
SRR26902955	BCA	12.3M	13.8M	0.00	0.00	0.00	0.00	0.00	0.00	0.00	0.00	0.00	1.00	5621.00	1,325,291.00	37,203.00
SRR26902956	BCA	12.0M	13.1M	0.00	0.00	0.00	0.00	0.00	0.00	0.00	0.00	0.00	0.00	1869.00	685,377.00	36,759.00
SRR26902957	BCA	14.1M	15.9M	0.00	0.00	0.00	0.00	0.00	0.00	0.00	0.00	0.00	0.00	9390.00	650,746.00	24,473.00
SRR26902958	BCA	12.1M	16.1M	0.00	1.00	0.00	0.00	0.00	0.00	0.00	0.00	0.00	0.00	3163.00	641,472.00	47,566.00
SRR26902959	BCA	13.1M	14.9M	0.00	1.00	0.00	0.00	0.00	0.00	0.00	0.00	0.00	0.00	5900.00	944,153.00	19,620.00
SRR26902960	BCA	14.7M	15.9M	0.00	0.00	0.00	0.00	0.00	0.00	0.00	0.00	0.00	0.00	4150.00	1,614,219.00	35,064.00
SRR26902961	BCA	10.6M	13.2M	0.00	0.00	0.00	0.00	0.00	0.00	0.00	0.00	0.00	0.00	19,870.00	932,350.00	14,936.00
SRR26902962	BCA	11.2M	15.1M	0.00	0.00	0.00	0.00	0.00	0.00	0.00	0.00	0.00	0.00	6504.00	1,201,883.00	14,219.00
SRR26902963	NEGATIVE	11.2M	14.3M	0.00	0.00	0.00	0.00	0.00	0.00	0.00	0.00	0.00	0.00	209.00	26,355.00	6327.00
SRR26902964	NEGATIVE	12.8M	13.5M	0.00	0.00	0.00	0.00	0.00	0.00	0.00	0.00	0.00	0.00	1409.00	122,413.00	36,674.00

^*a*^ BCA: breast cancer.

**TABLE 7:** PRJNA934049: Circulating miRNA biomarker for detecting BCA in high-risk benign breast tumors^*a*^^,*b*,*c*^

				Number of reads that align on												
Sample ID	Cancer type	Post trim reads	Total no of reads	blv-miR-B1-3p	blv-miR-B1-5p	blv-miR-B2-3p	blv-miR-B2-5p	blv-miR-B3-3p	blv-miR-B3-5p	blv-miR-B4-3p	blv-miR-B4-5p	blv-miR-B5-3p	blv-miR-B5-5p	hsa-miR-106b-5p	hsa-miR-21-5p	hsa-mir-29a-3p
SRR23425832	HRB	6.0M	9.3M	0.00	0.00	0.00	0.00	0.00	0.00	0.00	0.00	0.00	0.00	57.00	28,461.00	3807.00
SRR23425833	HRB	6.9M	9.4M	0.00	0.00	0.00	0.00	0.00	0.00	0.00	0.00	0.00	0.00	148.00	16,671.00	4156.00
SRR23425834	HRB	4.4M	7.5M	0.00	0.00	0.00	0.00	0.00	0.00	0.00	0.00	0.00	0.00	71.00	9938.00	1852.00
SRR23425835	HRB	5.4M	11.6M	0.00	0.00	0.00	0.00	0.00	0.00	0.00	0.00	0.00	0.00	33.00	9327.00	1320.00
SRR23425836	HRB	5.9M	8.8M	0.00	0.00	0.00	0.00	0.00	0.00	0.00	0.00	0.00	0.00	65.00	17,191.00	2073.00
SRR23425837	HRB	6.7M	11.3M	0.00	0.00	0.00	0.00	0.00	0.00	0.00	0.00	0.00	0.00	68.00	25,093.00	2631.00
SRR23425838	HRB	6.1M	9.2M	0.00	0.00	0.00	0.00	1.00	0.00	0.00	0.00	0.00	0.00	66.00	24,666.00	2331.00
SRR23425839	HRB	6.5M	10.2M	0.00	0.00	0.00	0.00	0.00	0.00	0.00	0.00	0.00	1.00	62.00	24,237.00	2600.00
SRR23425840	HRB	7.8M	12.6M	0.00	0.00	0.00	0.00	0.00	0.00	0.00	0.00	0.00	0.00	67.00	31,239.00	3107.00
SRR23425841	HRB	4.9M	9.8M	0.00	0.00	0.00	0.00	0.00	0.00	0.00	0.00	0.00	0.00	57.00	14,946.00	2709.00
SRR23425842	HRB	52.8M	92.6M	0.00	0.00	0.00	0.00	0.00	0.00	0.00	0.00	0.00	0.00	455.00	150,618.00	30,103.00
SRR23425843	HRB	5.6M	10.3M	0.00	0.00	0.00	0.00	0.00	0.00	0.00	0.00	0.00	0.00	54.00	19,832.00	3748.00
SRR23425844	HRB	6.3M	11.2M	0.00	0.00	0.00	0.00	0.00	0.00	0.00	0.00	0.00	0.00	126.00	46,332.00	9883.00
SRR23425845	HRB	6.0M	8.9M	0.00	0.00	0.00	0.00	0.00	0.00	0.00	0.00	0.00	0.00	25.00	21,386.00	1679.00
SRR23425846	HRB	5.8M	8.1M	0.00	0.00	0.00	0.00	0.00	0.00	0.00	0.00	0.00	3.00	26.00	8207.00	1701.00
SRR23425847	HRB	7.5M	8.9M	0.00	0.00	0.00	0.00	0.00	0.00	0.00	0.00	0.00	0.00	72.00	96,872.00	5920.00
SRR23425848	HRB	7.7M	9.9M	0.00	0.00	0.00	0.00	0.00	0.00	0.00	0.00	0.00	0.00	74.00	31,431.00	3323.00
SRR23425849	HRB	4.9M	9.1M	0.00	0.00	0.00	0.00	0.00	0.00	0.00	0.00	0.00	0.00	28.00	33,311.00	2283.00
SRR23425850	ESC	5.0M	8.9M	0.00	0.00	0.00	0.00	0.00	0.00	0.00	0.00	0.00	0.00	39.00	34,418.00	1967.00
SRR23425851	ESC	3.5M	8.7M	0.00	0.00	0.00	0.00	0.00	0.00	0.00	0.00	0.00	0.00	4.00	12,939.00	1223.00
SRR23425852	ESC	5.4M	8.6M	0.00	0.00	0.00	0.00	0.00	0.00	0.00	0.00	0.00	0.00	71.00	31,484.00	4186.00
SRR23425853	ESC	2.4M	8.5M	0.00	0.00	0.00	0.00	0.00	0.00	0.00	0.00	0.00	0.00	14.00	10,472.00	1332.00
SRR23425854	ESC	4.7M	7.8M	0.00	0.00	0.00	0.00	0.00	0.00	0.00	0.00	0.00	0.00	20.00	16,228.00	2676.00
SRR23425855	ESC	6.8M	7.8M	0.00	0.00	0.00	0.00	0.00	0.00	0.00	0.00	0.00	0.00	109.00	72,177.00	3692.00
SRR23425856	ESC	5.1M	8.6M	0.00	0.00	0.00	0.00	0.00	0.00	0.00	0.00	0.00	1.00	69.00	21,932.00	2039.00
SRR23425857	ESC	4.6M	8.6M	0.00	0.00	0.00	0.00	0.00	0.00	0.00	0.00	0.00	0.00	46.00	19,768.00	1985.00
SRR23425858	ESC	4.6M	7.4M	0.00	0.00	0.00	0.00	0.00	0.00	0.00	0.00	0.00	0.00	49.00	22,279.00	2133.00
SRR23425859	NRB	4.1M	7.2M	0.00	0.00	0.00	0.00	0.00	0.00	0.00	0.00	0.00	2.00	48.00	16,368.00	1670.00
SRR23425860	NRB	5.3M	8.9M	0.00	0.00	0.00	0.00	0.00	0.00	0.00	0.00	0.00	0.00	62.00	21,002.00	2427.00
SRR23425861	NRB	5.5M	8.3M	0.00	0.00	0.00	0.00	0.00	0.00	0.00	0.00	0.00	0.00	62.00	26,416.00	2841.00
SRR23425862	NRB	4.5M	7.5M	0.00	0.00	0.00	0.00	0.00	0.00	0.00	0.00	0.00	0.00	58.00	18,479.00	1847.00
SRR23425863	NRB	6.3M	10.8M	0.00	0.00	0.00	0.00	0.00	0.00	0.00	0.00	0.00	1.00	60.00	25,429.00	2642.00
SRR23425864	NRB	4.0M	8.3M	0.00	0.00	0.00	0.00	0.00	0.00	0.00	0.00	0.00	0.00	74.00	12,184.00	2121.00
SRR23425865	NRB	4.5M	9.9M	0.00	0.00	0.00	0.00	0.00	0.00	0.00	0.00	0.00	0.00	47.00	18,390.00	2630.00
SRR23425866	NRB	6.5M	8.6M	0.00	0.00	0.00	0.00	0.00	0.00	0.00	0.00	0.00	0.00	148.00	55,271.00	6275.00
SRR23425867	NRB	3.7M	11.7M	0.00	0.00	0.00	0.00	0.00	0.00	0.00	0.00	0.00	0.00	10.00	4482.00	945.00

^*a*^ NRB: no-risk benign.

^*b*^ HRB: high-risk benign.

^*c*^ ESC: early-stage breast cancer.

**TABLE 8:** PRJNA792999: tRF and tiRNA sequencing of BCA tissue samples and matched non-tumor adjacent tissues^*a*^

				Number of reads that align on												
Sample ID	Cancer type	Post trim reads	Total no of reads	blv-miR-B1-3p	blv-miR-B1-5p	blv-miR-B2-3p	blv-miR-B2-5p	blv-miR-B3-3p	blv-miR-B3-5p	blv-miR-B4-3p	blv-miR-B4-5p	blv-miR-B5-3p	blv-miR-B5-5p	hsa-miR-106b-5p	hsa-miR-21-5p	hsa-mir-29a-3p
SRR17374780	CONTROL	3.2M	7.5M	0.00	0.00	0.00	0.00	0.00	0.00	0.00	0.00	0.00	0.00	846.00	511,803.00	13,671.00
SRR17374781	BCA	5.6M	8.6M	0.00	0.00	0.00	0.00	0.00	0.00	0.00	0.00	0.00	0.00	1029.00	165,760.00	21,253.00
SRR17374782	BCA	5.6M	8.6M	0.00	0.00	0.00	0.00	1.00	0.00	0.00	0.00	0.00	0.00	1172.00	249,216.00	19,424.00
SRR17374783	CONTROL	3.5M	7.4M	0.00	0.00	0.00	0.00	0.00	0.00	0.00	0.00	0.00	0.00	1262.00	723,307.00	11,318.00
SRR17374784	CONTROL	4.1M	8.5M	0.00	0.00	0.00	0.00	0.00	1.00	0.00	0.00	0.00	0.00	1380.00	441,183.00	20,845.00
SRR17374785	CONTROL	3.4M	7.0M	0.00	0.00	0.00	0.00	0.00	0.00	0.00	0.00	0.00	0.00	864.00	212,775.00	10,398.00
SRR17374786	BCA	7.7M	11.4M	0.00	0.00	0.00	0.00	1.00	0.00	0.00	0.00	0.00	0.00	1236.00	209,167.00	19,403.00
SRR17374787	CONTROL	5.3M	8.0M	0.00	1.00	0.00	0.00	0.00	0.00	0.00	0.00	0.00	0.00	1078.00	178,186.00	30,266.00
SRR17374788	BCA	5.8M	11.3M	0.00	0.00	0.00	0.00	0.00	0.00	0.00	0.00	0.00	0.00	1150.00	161,677.00	18,856.00
SRR17374789	BCA	7.5M	11.0M	0.00	0.00	0.00	0.00	0.00	0.00	0.00	0.00	0.00	0.00	1409.00	289,567.00	27,057.00

^*a*^ BCA: breast cancer.

**TABLE 9:** PRJNA758408: Circulating exosomal miRNAs as predictive biomarkers of neoadjuvant chemotherapy response in BCA^*a*^

				Number of reads that align on												
Sample ID	Cancer type	Post trim reads	Total no of reads	blv-miR-B1-3p	blv-miR-B1-5p	blv-miR-B2-3p	blv-miR-B2-5p	blv-miR-B3-3p	blv-miR-B3-5p	blv-miR-B4-3p	blv-miR-B4-5p	blv-miR-B5-3p	blv-miR-B5-5p	hsa-miR-106b-5p	hsa-miR-21-5p	hsa-mir-29a-3p
SRR15658006	BCA	12.6M	24.7M	0.00	0.00	0.00	0.00	0.00	0.00	0.00	0.00	0.00	0.00	1.00	2281.00	954.00
SRR15658007	BCA	9.3M	12.1M	0.00	0.00	0.00	0.00	0.00	0.00	0.00	0.00	0.00	0.00	0.00	714.00	1239.00
SRR15658008	BCA	13.0M	24.3M	0.00	0.00	0.00	0.00	0.00	0.00	0.00	0.00	0.00	0.00	7.00	7433.00	2771.00
SRR15658009	BCA	14.8M	25.5M	0.00	2.00	0.00	0.00	0.00	0.00	0.00	0.00	0.00	0.00	50.00	49,045.00	5029.00
SRR15658010	BCA	12.0M	25.2M	0.00	0.00	0.00	0.00	0.00	0.00	0.00	0.00	0.00	0.00	14.00	14,352.00	3276.00
SRR15658011	BCA	11.8M	21.5M	0.00	0.00	0.00	0.00	0.00	0.00	0.00	0.00	0.00	0.00	14.00	19,317.00	5017.00
SRR15658012	BCA	13.3M	24.1M	0.00	0.00	0.00	0.00	0.00	0.00	0.00	5.00	0.00	0.00	0.00	3573.00	1463.00
SRR15658013	BCA	12.9M	19.1M	0.00	0.00	0.00	0.00	0.00	0.00	0.00	2.00	0.00	0.00	0.00	1333.00	785.00
SRR15658014	BCA	7.3M	17.1M	0.00	0.00	0.00	0.00	0.00	0.00	0.00	0.00	0.00	0.00	0.00	1682.00	5269.00
SRR15658015	BCA	9.5M	17.5M	0.00	0.00	0.00	0.00	0.00	0.00	0.00	0.00	0.00	0.00	2.00	4631.00	8741.00
SRR15658016	BCA	8.8M	20.7M	0.00	0.00	0.00	0.00	0.00	0.00	0.00	0.00	0.00	0.00	6.00	6250.00	3228.00
SRR15658017	BCA	9.7M	19.9M	0.00	0.00	0.00	0.00	0.00	0.00	0.00	0.00	0.00	0.00	20.00	10,140.00	3642.00
SRR15658018	BCA	10.9M	20.1M	0.00	0.00	0.00	0.00	0.00	0.00	0.00	0.00	0.00	0.00	59.00	38,933.00	8483.00
SRR15658019	BCA	12.2M	20.0M	0.00	0.00	0.00	0.00	0.00	0.00	0.00	0.00	0.00	0.00	52.00	63,037.00	10,393.00
SRR15658020	BCA	4.0M	5.6M	0.00	0.00	0.00	0.00	0.00	0.00	0.00	0.00	0.00	0.00	0.00	2473.00	2136.00
SRR15658021	BCA	16.6M	23.4M	0.00	0.00	0.00	0.00	0.00	0.00	0.00	0.00	0.00	0.00	14.00	5989.00	2084.00
SRR15658022	BCA	8.5M	18.0M	0.00	0.00	0.00	0.00	0.00	0.00	0.00	0.00	0.00	0.00	3.00	5398.00	3700.00
SRR15658023	BCA	11.3M	20.0M	0.00	0.00	0.00	0.00	0.00	0.00	0.00	0.00	0.00	0.00	49.00	10,207.00	7480.00
SRR15658024	BCA	9.5M	17.3M	0.00	0.00	0.00	0.00	0.00	0.00	0.00	0.00	0.00	0.00	68.00	15,731.00	6682.00
SRR15658025	BCA	11.3M	20.7M	0.00	0.00	0.00	0.00	0.00	0.00	0.00	0.00	0.00	0.00	41.00	12,592.00	7573.00
SRR15658026	BCA	11.1M	19.7M	0.00	0.00	0.00	0.00	0.00	0.00	0.00	0.00	0.00	0.00	50.00	27,748.00	13,484.00
SRR15658027	BCA	10.3M	18.6M	0.00	0.00	0.00	0.00	0.00	0.00	0.00	0.00	0.00	0.00	3.00	7453.00	5892.00
SRR15658028	BCA	11.1M	19.6M	0.00	0.00	0.00	0.00	0.00	0.00	0.00	11.00	0.00	0.00	34.00	21,262.00	8018.00
SRR15658029	BCA	7.9M	16.4M	0.00	0.00	0.00	0.00	0.00	0.00	0.00	0.00	0.00	0.00	21.00	8610.00	4560.00
SRR15658030	BCA	8.7M	17.7M	0.00	0.00	0.00	0.00	0.00	0.00	0.00	0.00	0.00	0.00	48.00	7291.00	3855.00
SRR15658031	BCA	14.2M	20.3M	0.00	0.00	0.00	0.00	0.00	0.00	0.00	0.00	0.00	0.00	45.00	39,215.00	21,751.00
SRR15658032	BCA	12.4M	20.4M	0.00	0.00	0.00	0.00	0.00	0.00	0.00	0.00	0.00	0.00	104.00	40,141.00	11,822.00
SRR15658033	BCA	10.1M	18.9M	0.00	0.00	0.00	0.00	0.00	0.00	0.00	0.00	0.00	0.00	3.00	10,767.00	9387.00
SRR15658034	BCA	10.1M	19.6M	0.00	0.00	0.00	0.00	0.00	0.00	0.00	0.00	0.00	0.00	18.00	23,834.00	4825.00
SRR15658035	BCA	10.3M	18.7M	0.00	0.00	0.00	0.00	0.00	0.00	0.00	0.00	0.00	0.00	5.00	15,177.00	3724.00
SRR15658036	BCA	14.3M	20.8M	0.00	0.00	0.00	0.00	0.00	0.00	0.00	0.00	0.00	0.00	54.00	73,731.00	24,589.00
SRR15658037	BCA	10.5M	22.2M	0.00	0.00	0.00	0.00	0.00	0.00	0.00	0.00	0.00	0.00	5.00	8502.00	3850.00
SRR15658038	BCA	10.6M	22.3M	0.00	0.00	0.00	0.00	0.00	0.00	0.00	0.00	0.00	0.00	14.00	6727.00	3365.00
SRR15658039	BCA	11.6M	21.7M	0.00	0.00	0.00	0.00	0.00	0.00	0.00	0.00	0.00	0.00	22.00	8220.00	4864.00
SRR15658040	BCA	12.1M	24.4M	0.00	0.00	0.00	0.00	0.00	0.00	0.00	0.00	0.00	0.00	43.00	8446.00	4863.00
SRR15658041	BCA	9.8M	18.8M	0.00	0.00	0.00	0.00	0.00	0.00	0.00	0.00	0.00	0.00	0.00	21,313.00	5522.00
SRR15658042	BCA	9.1M	19.6M	0.00	0.00	0.00	0.00	0.00	0.00	0.00	0.00	0.00	0.00	14.00	8176.00	5296.00
SRR15658043	BCA	11.0M	20.4M	0.00	0.00	0.00	0.00	0.00	0.00	0.00	0.00	0.00	0.00	18.00	11,067.00	5029.00
SRR15658044	BCA	6.1M	9.5M	0.00	0.00	0.00	0.00	0.00	0.00	0.00	0.00	0.00	0.00	0.00	1704.00	4615.00
SRR15658045	BCA	9.4M	18.8M	0.00	0.00	0.00	0.00	0.00	0.00	0.00	0.00	0.00	0.00	3.00	4907.00	5203.00

^*a*^ BCA: breast cancer.

## RESULTS

We analyzed a total of 335 human samples from nine different small RNAseq studies using the Galaxy.org platform. A total of 228 cancer samples: BCA, ALL, AML, CLL, and 107 controls were tested for the presence of BLV-miRNAs. A total of 1.98 billion trimmed reads (3.1 billion raw) from human cancer and control data sets were analyzed. Only 60 reads (0.000003%) aligned to BLV miRNA reference sequences, most mapping to STAR strands and/or containing up to two mismatches (Tables 1 to 9). For example, SRR15658028 (PRJNA758408), which had 11 reads mapping to blv-miR-B4-5p STAR strands, had two substitutions compared to the reference sequence (Fig. S1). The detection of human miRNA controls confirmed data quality across most data sets, except for PRJNA996975, which was excluded due to low sequencing depth and poor recovery of internal positive controls.

Additionally, a subset of three small RNA-seq data sets from white blood cells of cattle infected and three from cattle non-infected with BLV were analyzed to validate the analytical workflow in biological samples. In cattle data sets, 9 of 10 BLV miRNAs were robustly detected in BLV-positive samples, validating our workflow (Table 10). BLV miRNAs were detected at lower frequencies in BLV-uninfected cattle and were attributed to background noise or early infection stages. Normalized abundance (RPM) confirmed a >200-fold difference between infected and uninfected cattle (10669 vs. 42 RPM). These results demonstrate that the pipeline can reliably detect BLV miRNAs when present in biological samples.

**TABLE 10:** Number of normalized counts aligned on BLV miRNAs (cattle sRNA-seq)^*a*^

BLV status	Animal ID	Total number of reads	NORMALIZED COUNTS that align on (RPM)									
			blv-miR-B1-3p	blv-miR-B1-5p	blv-miR-B2-5p	blv-miR-B2-3p	blv-miR-B3-5p	blv-miR-B3-3p	blv-miR-B4-3p	blv-miR-B4-5p	blv-miR-B5-5p	blv-miR-B5-3p
BLV-infected	SRR5659715	14,100,000	5224	30	889	1344	32	314	1732	0	31	100
	SRR5659721	12,700,000	12	1	3	6	0	2	6	0	0	1
	SRR5659722	10,700,000	313	8	141	255	8	55	113	0	14	33
BLV-uninfected	SRR5659714	8,400,000	6	0	1	2	0	0	2	0	0	0
	SRR5659718	18,700,000	10	0	1	3	0	1	4	0	0	0
	SRR5659719	17,400,000	5	0	1	2	0	0	2	0	0	0

^*a*^ RPM: reads per million of mapped reads.

## DISCUSSION

Several viruses are known to contribute to the development of human malignancies (37); however, it is not clear whether BLV exposure might augment the risk of BCA in humans (2, 38). While some studies support this hypothesis (3–13), other studies argue against it (14–16). In previous studies, whole-genome and whole-transcriptome sequencing analysis of human BCA samples failed to detect sequencing reads corresponding to BLV proviral DNA or viral transcripts in these samples (15, 16). This study explored an alternative viral factor potentially involved in oncogenesis. BLV encodes a set of miRNAs that are highly expressed during natural BLV infection in cattle (22, 39), and some of these miRNAs could interact with human tumoral suppressor genes (19, 20). Interestingly, bovine miRNAs can be conveyed through EVs present in milk from cattle (25, 33, 40). Moreover, EVs containing miRNAs are absorbed in the intestine and subsequently transferred into the bloodstream of the individuals who ingest them (29–31). Different human cells (i.e., hepatic cells, macrophages, PBMCs) can incorporate exogenous EVs (30, 32, 41), and miRNAs within them can regulate gene expression (30). Remarkably, EVs containing miRNAs are resistant to pasteurization, and miRNAs contained in different commercial dairy products (i.e., milk, baby formula milk, etc.) can be absorbed by humans (26, 27, 30, 32, 40). Taken together, these observations allowed us to hypothesize that there could be an association between BLV miRNAs and particular human cancers, even in the absence of BLV exposure.

In this study, we screened 335 publicly available human small-RNA sequencing data sets from BCA, leukemias, and healthy controls to determine whether BLV-encoded miRNAs could be detected in human cancer miRNomes. This hypothesis was motivated by the established oncogenic role of BLV miRNAs in cattle and the ongoing controversy regarding potential BLV exposure, infection, or dietary transfer in humans. Rather than testing causation directly, our objective was to perform a sensitive empirical screen to determine whether any reproducible BLV miRNA signal was detectable in human cancer data sets.

Across 1.98 billion trimmed human reads, only 60 reads aligned permissively to BLV miRNA references, predominantly representing STAR sequences or containing mismatches. These patterns are consistent with expected statistical noise under high-sensitivity alignment conditions rather than genuine biological detection. In contrast, our analysis of BLV-infected cattle data sets robustly detected 9 out of 10 canonical BLV miRNAs, confirming that the analytical workflow can identify authentic BLV miRNAs when present at biologically relevant levels. We therefore interpret the sparse BLV-like alignments in human samples as background noise.

As internal quality controls, we evaluated three human miRNAs linked to oncogenic processes (hsa-miR-29a-3p, hsa-miR-106b-5p, and hsa-miR-21-5p) (21, 42). Their consistent detection across data sets (except PRJNA996975, excluded due to low read depth and impaired recovery of internal controls) demonstrates that the analyzed libraries were suitable for miRNA detection. Together with the high BLV miRNA levels observed in BLV-infected cattle, these results validate both the sensitivity of our workflow and the integrity of the human data sets.

However, several methodological limitations must be acknowledged. First, the data sets analyzed predominantly consist of bulk tumor or peripheral blood sRNA-seq libraries, which may not correspond to biological compartments aligned with proposed exposure models. If the presence of BLV miRNAs in humans arises from direct viral infection, they might localize primarily to lymphoid-associated compartments rather than bulk tumor tissue. In addition, BLV may replicate in cell types distinct from those infected in the natural host. In this regard, Moran and colleagues demonstrated the ability of BLV to infect human mammary epithelial cells (43). Alternatively, if derived through dietary EV transfer, detection may require postprandial plasma sampling, when EV concentrations peak. Thus, our inference applies specifically to the sampled compartments and timepoints represented in the public data sets analyzed. Second, we did not establish quantitative detection limits through matrix-matched synthetic spike-ins processed using the same sequencing workflows. Accordingly, “below detection threshold” reflects the operational sensitivity of this pipeline rather than absolute biological absence. Third, we relied on canonical miRBase BLV references, which may not capture divergent BLV genotypes or non-canonical isomiRs potentially relevant to human exposure. Future analyses incorporating explicit false-positive benchmarking, including competitive decoy alignments and parameter sweeps to quantify mapping specificity under high-sensitivity settings, will help distinguish true low-level signals from alignment noise. Variant-aware mapping and competitive alignment strategies against the full human miRNAome/genome may further improve specificity and reduce cross-mapping artifacts, particularly given the shared seed sequences between blv-miR-b4-3p and hsa-miR-29a. Additionally, because the data sets analyzed were not optimized for rare viral miRNA detection, targeted enrichment strategies—such as argonaut immunoprecipitation (AGO-IP), locked nucleic acid (LNA)-based capture probes, or viral hairpin pull-down—may be required during future library preparations to detect extremely low-abundance or transient molecules. AGO-IP enriches for miRNAs actively loaded into the RISC complex, whereas LNA-based capture and viral hairpin pull-down selectively enrich predefined viral miRNAs and precursor miRNAs, respectively. Together, these approaches enhance sensitivity for detecting low-abundance or transient viral miRNAs.

In summary, within the limitations of publicly available small-RNA data sets, canonical reference alignments, and standard sequencing workflows, we found no evidence supporting the presence of BLV miRNAs in human BCA and human leukemia samples. While trace or transient signals cannot be entirely excluded, the data indicate that BLV miRNAs are not a reproducible feature of human cancer miRNomes. These findings contribute empirical clarity to a longstanding controversy and highlight the need for future research incorporating targeted enrichment, spike-in calibration, variant-aware reference sets, and compartment- or time-specific sampling to definitively evaluate mechanistic hypotheses of BLV exposure in humans.

## Supplementary Material

Reviewer comments

## SUPPLEMENTAL MATERIAL

Figure S1 spectrum.03818-25-s0001.docx Alignment of sRNA seq reads from human cancer sample SRR15658028 (PRJNA758408) on BLV miRNAs.

Table S1 spectrum.03818-25-s0002.docx Reference miRNA.

OPEN PEER REVIEW reviewer-comments.pdf An accounting of the reviewer comments and feedback.

ASM does not own the copyrights to Supplemental Material that may be linked to, or accessed through, an article. The authors have granted ASM a non-exclusive, world-wide license to publish the Supplemental Material files. Please contact the corresponding author directly for reuse.
