# Supplementary material for: Absence of detectable bovine leukemia virus miRNAs in human cancer small RNA-seq datasets
Source: Microbiol Spectr. 2026 Mar 16;14(4):e03818-25. doi: 10.1128/spectrum.03818-25 (PMC13055368; doi:10.1128/spectrum.03818-25)
Supplement: Figure S1 — Alignment of sRNA seq reads from human cancer sample SRR15658028 (PRJNA758408) on BLV miRNAs. [file spectrum.03818-25-s0001.docx]

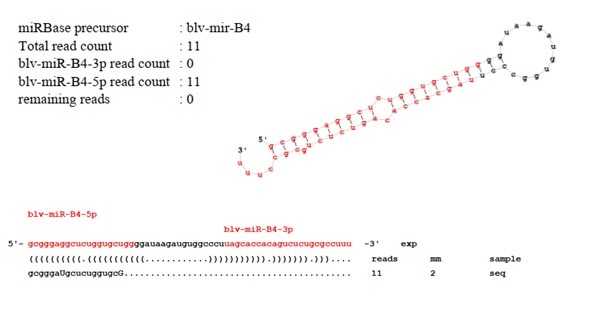


**Supplementary Figure 1**. Alignment of sRNA seq reads from human cancer sample SRR15658028 (PRJNA758408) on BLV miRNAs. This is a representative sample from a total of 27 human cancer samples that aligned on BLV miRNAs (total 60 raw reads). Because of using a “highly sensitive” miRDeep2 quantifier tool setting, some reads aligned to BLV miRNA STAR sequences and presented up to two mismatches compared to BLV miRNA reference sequences.
