# Supplementary material for: Absence of detectable bovine leukemia virus miRNAs in human cancer small RNA-seq datasets
Source: Microbiol Spectr. 2026 Mar 16;14(4):e03818-25. doi: 10.1128/spectrum.03818-25 (PMC13055368; doi:10.1128/spectrum.03818-25)
Supplement: Table S1 — Reference miRNA. [file spectrum.03818-25-s0002.docx]

| Supplementary table 1. Reference miRNA |
| --- |
|  |
| **Precursor miRNAs** |
| >hsa-mir-29a |
| ATGACTGATTTCTTTTGGTGTTCAGAGTCAATATAATTTTCTAGCACCATCTGAAATCGGTTAT |
| >hsa-miR-106b |
| CCTGCCGGGGCTAAAGTGCTGACAGTGCAGATAGTGGTCCTCTCCGTGCTACCGCACTGTGGGTACTTGCTGCTCCAGCAGG |
| >hsa-miR-21 |
| TGTCGGGTAGCTTATCAGACTGATGTTGACTGTTGAATCTCATGGCAACACCAGTCGATGGGCTGTCTGACA |
| >blv-mir-B1 |
| AGGCTGTGGTGGTGCACTGGCTTAGTGGAGTAGTCAGTGTACCATCACAAGCCTCT |
| >blv-mir-B2 |
| ATGACTGAGTGTAGCGCAGAGAGATTGTCGCTTCTGCGTGTCGCTCAGTCATTTT |
| >blv-mir-B3 |
| ATCCCCCTGCCAGCGTTGGTCTAGTGGAAAGAACTAACGCTGACGGGGGCGATTTCT |
| >blv-mir-B4 |
| GCGGGAGGCTCTGGTGCTGGGGATAAGATGTGGCCCTTAGCACCACAGTCTCTGCGCCTTT |
| >blv-mir-B5 |
| AGGAAGGTTGTGGCTCAGAGGTTAAAATAGCTCGAGCCGCAACCTCCCTTTCT |
|  |
| **Mature miRNAs** |
| >hsa-mir-29a-3p |
| TAGCACCATCTGAAATCGGTTA |
| >hsa-miR-106b-5p |
| TAAAGTGCTGACAGTGCAGAT |
| >hsa-miR-21-5p |
| TAGCTTATCAGACTGATGTTGA |
| >blv-miR-B1-3p |
| TCAGTGTACCATCACAAGCCTCT |
| >blv-miR-B2-5p |
| ATGACTGAGTGTAGCGCAGAGA |
| >blv-miR-B2-3p |
| TGCGTGTCGCTCAGTCATTTT |
| >blv-miR-B3-5p |
| ATCCCCCTGCCAGCGTTGGTC |
| >blv-miR-B3-3p |
| TAACGCTGACGGGGGCGATTTCT |
| >blv-miR-B4-3p |
| TAGCACCACAGTCTCTGCGCCTTT |
| >blv-miR-B5-5p |
| AGGAAGGTTGTGGCTCAGAGGT |
| >blv-miR-B5-3p |
| CTCGAGCCGCAACCTCCCTTTCT |
| >blv-miR-B1-5p |
| AGGCTGTGGTGGTGCACTGGCTT |
| >blv-miR-B4-5p |
| GCGGGAGGCTCTGGTGCTGG |
